# Role of Mitochondria in the Mechanism(s) of Action of Metformin

**DOI:** 10.3389/fendo.2019.00294

**Published:** 2019-05-07

**Authors:** Guillaume Vial, Dominique Detaille, Bruno Guigas

**Affiliations:** ^1^Laboratoire Hypoxie-Physiopathologies Cardiovasculaires et Respiratoires HP2, Faculté de Médecine et de Pharmacie, INSERM U1042, La Tronche, France; ^2^Laboratoire Hypoxie-Physiopathologies Cardiovasculaires et Respiratoires HP2, Faculté de Médecine et de Pharmacie, Université Grenoble-Alpes, La Tronche, France; ^3^Centre de Recherche Cardio-Thoracique de Bordeaux, INSERM U1045, Université de Bordeaux, Bordeaux, France; ^4^Department of Parasitology, Leiden University Medical Center, Leiden, Netherlands

**Keywords:** biguanides, respiratory-chain complex 1, bioenergetics, AMPK, cancer

## Abstract

Metformin is a drug from the biguanide family that is used for decades as the first-line therapeutic choice for the treatment of type 2 diabetes. Despite its worldwide democratization, owing to its clinical efficacy, high safety profile and cheap cost, the exact mechanism(s) of action of this anti-hyperglycemic molecule with pleiotropic properties still remains to be fully elucidated. The concept that metformin would exert some of its actions though modulation of the mitochondrial bioenergetics was initially forged in the 50s but undeniably revived at the beginning of the twenty-first century when it was shown to induce a weak but specific inhibition of the mitochondrial respiratory-chain complex 1. Furthermore, metformin has been reported to reduce generation of reactive oxygen species at the complex 1 and to prevent mitochondrial-mediated apoptosis, suggesting that it can protect against oxidative stress-induced cell death. Nevertheless, despite some recent progress and the demonstration of its key role in the inhibition of hepatic gluconeogenesis, the exact nature of the mitochondrial interaction between the drug and the complex 1 is still poorly characterized. Recent studies reported that metformin may also have anti-neoplastic properties by inhibiting cancer cell growth and proliferation, at least partly through its mitochondrial action. As such, many trials are currently conducted for exploring the repositioning of metformin as a potential drug for cancer therapy. In this mini-review, we discuss both historical and more recent findings on the central role played by the interaction between metformin and the mitochondria in its cellular mechanism of action.

## Introduction

Historically, the origins of metformin (dimethylbiguanide) came from the Middle Age where medieval doctors used extract from the French Lilac *Galega officinalis* to treat various diseases (1). At the beginning of the twentieth century, the plant was found to be rich in guanidine, an active ingredient that was later reported to have potent anti-hyperglycemic properties. Guanidine derivatives gave rise to the biguanide family, among which metformin is currently the only therapeutic survivor for the treatment of type 2 diabetes. Indeed, after withdrawal of buformin and phenformin at the end of the 70′s, metformin hydrochloride gradually became the most widely prescribed oral antidiabetic agent, due to its efficient glucose-lowering effect, weight-neutral characteristic, high safety profile associated with low risk of hypoglycemia, and cost-effectiveness as a generic drug (2). Since then, metformin is well recognized for its ability to lower hyperglycemia by decreasing hepatic glucose production while reducing glucotoxicity in different tissues, a feature that might explain some of its cardioprotective benefits (2, 3). However, despite its worldwide democratization, the exact mechanism(s) of action of this molecule with apparent pleiotropic properties still remains to be fully elucidated. As many drugs, the cellular effects of metformin rely on its unique physicochemical characteristics, which include a high hydrophilicity, some metal-binding properties and a pKa within the physiological pH range, implying that the molecule exists solely in its positively charged cationic form (4). Due to its poor lipophilicity, metformin does not cross cell membranes by simple passive diffusion and its bio-distribution relies on tissue-specific transporters, including plasma membrane monoamine transporter (PMAT) in the intestine, organic cation transporter 1 (OCT1) in the liver, and both organic cation transporter 2 (OCT2) and multidrug and toxin extruder (MATE)1/2 in the kidneys (4, 5). By contrast, phenformin exhibits a higher lipophilicity than metformin, owing to its larger phenylethyl side chain, and is therefore crossing more easily lipid membrane bilayer, a property that might explain their differences in terms of selectivity and potency. Various underlying mechanisms have been suggested for metformin throughout the six decades following its first commercialization but a consensus only started to emerge during the last years, placing mitochondria at the heart of metformin's cellular actions.

## The Mitochondrial Respiratory-Chain Complex 1 as Primary Target of Metformin

**Figure 1:**
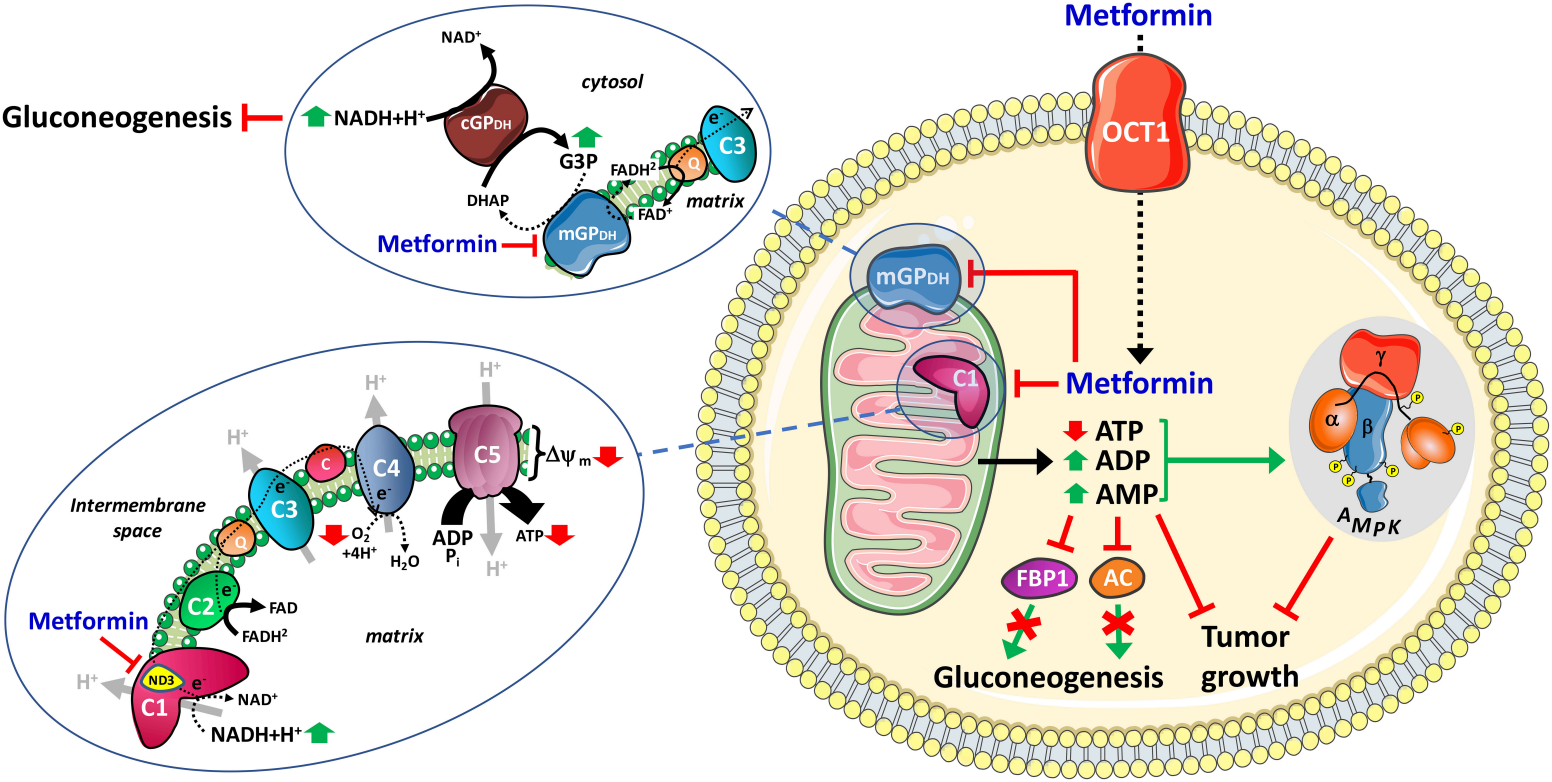
Mitochondrial mechanisms of action of metformin. After cellular uptake, mainly through OCT1 in hepatocytes, the mitochondria is the primary target of metformin which exerts specific inhibition on the respiratory-chain complex 1, presumably through direct interaction with the ND3 core subunit, and on mitochondrial glycerophosphate dehydrogenase (mGP_DH_). The inhibition of complex 1 decreases NADH oxidation, proton pumping across the inner mitochondrial membrane and oxygen consumption rate, resulting in lower proton gradient (Δψ) and reduction of proton-driven ATP synthesis from ADP and inorganic phosphate (Pi). The inhibition of mGP_DH_ modulates cytosolic and mitochondrial redox state resulting in increased cytosolic NADH. FBP1, fructose-1,6-bisphosphatase-1. AC, adenylate cyclase.

At the beginning of 2000, the group of Xavier Leverve was the first to report that metformin selectively inhibits the mitochondrial respiratory-chain complex 1 and, as a result, decreases NADH oxidation, reduces proton gradient across the inner mitochondrial membrane, and decreases oxygen consumption rate (6) (Figure 1). This major breakthrough was rapidly complemented by a supportive study from Halestrap's group published couple of months later (7). Although the inhibitory effect of metformin on complex 1 was first evidenced in rat hepatocytes in these two seminal studies, it was thereafter confirmed in various species and plenty of biological models, including lately in cancer cells (Table 1). Importantly, metformin only exerts a weak and reversible selective inhibition of complex 1 (IC_50_ ~20 mM), making it a peculiar type of inhibitor that does not resemble the canonical ones like rotenone and piericidin A (IC_50_ ~2 μM), which are both uncharged and highly hydrophobic molecules (24). It is worth mentioning that, although the discovery of complex 1 inhibition by metformin undoubtedly constituted a major advance in the understanding of its cellular mode of action, some inhibitory effects of biguanides on mitochondrial oxidative phosphorylation (OXPHOS) were already reported by Gunnar Hollunger, a Swedish scientist at the University of Lund, as early as 1955 (25), and by the German biochemist Günter Schäfer in the 60's (26).

**Table 1:** *Ex vivo* and *in vitro* mitochondrial effects of metformin.

	**Cell type**	**Metformin** **(mM)**	**Duration**	**Effects**	**Reference**
Healthy cells/organelles	Primary rat hepatocytes	0.1–10	45 min	Inhibition of *J*O_2_ Inhibition of C1-linked m*J*O_2_	(6)
		0.05	2 h	Non-competitive inhibition of mGPDH activity Inhibition of mGPDH-linked *J*O_2_	(8)
	Mouse hepatocytes	5	30 min	Inhibition of *J*O_2_ Inhibition of C1-linked m*J*O_2_	(9)
	Human hepatocytes	5	30 min	Inhibition of *J*O_2_ Inhibition of C1-linked m*J*O_2_	(9)
	*Xenopus laevis* oocytes	0.05	4–18 h	Inhibition of C1	(10)
		10	0.5–3 h	Inhibition of C1	(10)
	Rat liver mitochondria	0.5–5	1 min	No effect	(11)
		5–20	?	Inhibition of C1-linked *J*O_2_	(8)
		8–10	1 min	Inhibition of *J*O_2_ and RCR Decrease in ΔΨm	(11)
		10	30 min	No effect	(6)
		2–11	5 min	Inhibition of C1-linked *J*O_2_ Decrease in NADH oxidation	(12)
		>10	5 min	No effect	(7)
		1–10	4h (cold)	Inhibition of *J*O_2_ Inhibition of C1-linked *J*O_2_	(7)
	Mouse muscle mitochondria	2–5	30 min	Inhibition of C1-linked *J*O_2_ Inhibition of TCA cycle activity	(13)
	*Xenopus laevis* mitochondria	0.05–10	3 h	No effect	(10)
	Rat/liver heart SMPs	5–50	Immediate	Inhibition of C1 activity	(7)
	Bovine heart SMPs	100	Immediate	Inhibition of NADH oxidation	(14)
Cancer cells	Rat hepatoma H4IIE cells	0.05–0.1	24–60 h	Inhibition of C1-linked m*J*O_2_	(7)
		2	2 h30	Inhibition of *J*O_2_	(12)
	Mouse breast NT2196 cells	0.5–5	24–48 h	Inhibition of *J*O_2_ Increase in uncoupled *J*O_2_	(13)
	Human liver hepatoma HepG2 cells	2	0.5–8 h	Inhibition of *J*O_2_	(14)
	Human oral squamous carcinoma KB cells	0.1–10	0.5–24 h	Inhibition of *J*O_2_ Inhibition of C1-linked m*J*O_2_ Inhibition of isolated C1 activity	(15)
	Human colorectal HCT116, prostate LNCaP, squamous SCC-74B and colon POP-092S carcinoma cells	0.2–10	1–8 h	Inhibition of *J*O_2_	(16)
	Human breast MCF7 cells	0.5–5	24 h	Inhibition of *J*O_2_ Increase in uncoupled *J*O_2_ Inhibition of TCA cycle activity	(13)
		2.5–5	5 h	No effect on *J*O_2_ Inhibition of CYP3A4 AA Epoxygenase activity	(17)
	Human thyroid FTC133 and BCAP carcinoma cells	5	48 h	Inhibition of *J*O_2_ Lower mGPDH expression	(18)
		1–5	10 min	Inhibition of mGPDH activity	(18)
	Human lung A549 and cervical HeLa carcinoma cells	1	5–10 min	Inhibition of C1-linked m*J*O_2_ No effect on mG3PDH-linked m*J*O_2_	(19)
	Human pancreatic PDAC stem cells	3–10	1 h	Inhibition of *J*O_2_ Inhibition of C1-linked m*J*O_2_	(20)
	Human HCT116 p53^−/−^ colorectal carcinoma cells	0.25–1	24 h	Inhibition of *J*O_2_ Inhibition of C1-linked m*J*O_2_	(21)
	Human pancreatic PANC-1 carcinoma cells	0.5–10	48 h	Inhibition of C1-linked m*J*O_2_	(22)
		1–10	24 h	Inhibition of *J*O_2_ Inhibition of C1-linked m*J*O_2_	(23)
	Human pancreatic MiaPaCa-2 carcinoma cells	1–10	24 h	Inhibition of *J*O_2_ Inhibition of C1-linked m*J*O_2_	(23)

*C1, mitochondrial respiratory-chain complex 1; mG3P_DH_,mitochondrial glycerol-3-phosphate dehydrogenase; JO_2_, oxygen consumption rate; mJO_2_, mitochondrial oxygen consumption rate; RCR, respiratory control ratio; ROS, reactive oxygen species; TCA, tricarboxylic acid; SMPs, sub-mitochondrial particles*.

### Targeting the Mitochondria for Selective Inhibition of Mitochondrial Complex 1

How exactly metformin gets into the mitochondria and whether it inhibits complex 1 directly or not remains unclear and is still a matter of debate (27). Very high concentrations of metformin (20–100 mM) were reported to directly inhibit complex 1 activity in isolated mitochondria or in inside-out structured sub-mitochondrial particles (SMPs) whereas clinically relevant drug concentrations (<100 μM) did not (Table 1). By contrast, micromolar concentrations of the drug are sufficient to achieve a dose- and time-dependent *in situ* inhibition of mitochondrial complex 1 in various cell types (6, 10, 15, 28, 29) or *in vivo* in skeletal muscle from healthy and diabetic rats (30). Among the possible explanations, the positive charge of metformin was proposed to account for its slow accumulation within the matrix of energized mitochondria of intact cells, driven by their transmembrane electrochemical potential (ΔΨ) (7, 14). Indeed, according to thermodynamic laws and the Nernst equation, a ~1,000-fold accumulation of a positively charged molecule might theoretically occur in energized mitochondria with a physiologically relevant ΔΨ, suggesting that metformin could reach millimolar concentration in the organelle despite a cytoplasmic level within the micromolar range (27). Furthermore, a ΔΨ-driven mitochondrial import of the biguanide might also provide an explanation for its weak inhibitory effect on complex 1, the reduction in mitochondrial membrane potential induced by the drug limiting its subsequent buildup. However, no accumulation of radioactively-labeled metformin was observed in mitochondria isolated from *Xenopus laevis* oocytes and exposed to concentrations of the drug that inhibit complex 1 (10). Therefore, even if a direct effect of metformin on complex 1 turns out possible, it seems to be highly facilitated in intact cells regardless of the exact mechanism involved in this process. Although the low accumulation of metformin into mitochondria could primarily be explained by the slow permeation of the drug across the plasma membrane, some studies have also suggested the existence of a specific transport system mediating its mitochondrial import. As such, the observation that the inhibitory effect of metformin on complex 1 was temperature-dependent in intact *Xenopus laevis* oocytes and that low concentration (50 μM) was able to directly inhibit complex 1 activity in isolated mitochondria when delivered as a liposomal-encapsulated form that can eventually fuse with the organelle led to the hypothesis of an endocytic vesicular routing of the drug from the plasma membrane to the mitochondria (10). However, the molecular components involved in this putative process still remain obscure and would deserve extensive investigation, including in mammalian cells. On the other hand, it has also been reported that intra-mitochondrial accumulation of phenformin, another biguanide, could at least partly be mediated by the mitochondrial organic cation/carnitine transporter 1 (OCTN1) (31). More recently, a protein-mediated mitochondrial import of the biguanide was also suggested based on the fact that direct conjugation of a phenyl group and bis-substitution of the biguanide moiety on the molecule prevent its uptake into mitochondria, irrespective of the compound hydrophobicity (32). However, whether this could also occur for metformin was not assessed and remains therefore to be investigated.

### Molecular Interaction Between Metformin and the Respiratory-Chain Complex 1

The mammalian respiratory-chain complex 1 (NADH:ubiquinone oxidoreductase) is a large L-shaped membrane-bound redox enzyme composed of at least 45 different subunits that couples the transfer of electrons from NADH to the ubiquinone pool with a transfer of protons from the mitochondrial matrix toward the intermembrane space (33). The complex 1 exists in two distinct forms: a fully competent active one and a so-called “deactive” D-form where the enzyme is catalytically incompetent but can be activated by a slow reaction of NADH oxidation coupled to ubiquinone reduction (34). As metformin inhibits NADH oxidation by complex 1 in isolated mitochondria from bovine heart, yeast *Pichia pastoris*, bacterium *Escherichia coli* (14, 32), as well as from *C. elegans* (35), it is likely that the molecule binds to some of the phylogenetically conserved “core” subunits of the complex rather than to mammalian-specific accessory ones (33). While it has been shown that metformin did not alter the structural integrity of the whole complex (14), the exact molecular interactions between the drug and the complex 1 remain to be elucidated. In order to investigate how metformin, and other biguanides, could interact with complex 1 for regulating its activity, the group of Judy Hirst has elegantly dissected the effects of the drug at different levels of the catalytic cycle of the enzyme. They demonstrated that metformin is a reversible non-competitive inhibitor that probably binds to some amphipathic regions of the enzyme, i.e., where some hydrophilic and hydrophobic amino acids are in close proximity, and inhibits a rate-limiting step coupled to ubiquinone reduction, but does not competitively bind to the ubiquinone-binding site on complex 1 (14). Moreover, metformin rather stimulates the NADH:FeCN oxidoreduction reaction and does not alter the thermal stability of the flavin site, except at extremely high non-relevant concentration (200 mM), indicating that NADH oxidation occurring at the flavin site is probably not involved in the inhibition of complex 1 by the drug. Similarly, metformin does not modulate the FeS cluster of the NADH-reduced complex 1, suggesting that the intramolecular electron transfer is not impaired (14). However, using SMPs, the authors showed that inhibition of NADH oxidation by metformin is immediate when the drug is added prior to the initiation of catalysis but is delayed once catalysis has already started (14). Altogether, this strongly suggests that the inhibition depends on the catalytic status of complex 1, occurring primarily when the enzyme is in its “deactive” conformation with redox and proton transfer domains no longer efficiently coupled (14). Ultimately, the authors proposed that the Cys39-containing matrix loop of subunit ND3 located within the amphipathic region between the redox and proton-transfer domains might be the binding site for metformin on complex 1, stabilizing the enzyme in an open-loop deactive conformation state (14). It is worth mentioning that most of the above-mentioned mechanistic studies were performed using isolated organelles and high concentrations of the drug and that such experimental *in vitro* conditions might not always reflect the physiological *in situ* environment. For instance, complex 1 forms respiratory-chain supercomplexes together with complexes 3 and 4 (36), a supramolecular organization that is lost in SMPs and may affect the interaction of metformin with complex 1 and/or the regulatory effect of the drug on mitochondrial OXPHOS.

### Modulation of ROS Production at Complex 1 by Metformin

Besides their central role in cellular energy homeostasis, mitochondria are also the main source of reactive oxygen species (ROS) which, on top of potentially causing oxidative damages, could also play a key role as signaling molecules in various pathways (37). Superoxide anions are primarily generated by the mitochondria, mostly at complexes 1 and 3 of the electron transfer chain (ETC) where electrons are leaking and could react with oxygen. It is now well recognized that complex 1 can produce superoxide by both forward (site I_F_) and reverse electron (site I_Q_) fluxes, depending on substrates used to fuel the ETC (38). As such, rotenone can either increase or decrease mitochondrial ROS production at complex 1, depending on whether glutamate-malate (forward) or succinate (reverse) are provided as respiratory-chain substrates, respectively. By contrast, it has been shown that metformin specifically decreases the ROS production driven by the reverse electron transfer (RET) but without increasing ROS generation through the forward direction (39). Interestingly, a similar lowering effect on ROS production at the complex 1 than the one observed with metformin was also recently reported for imeglimin, a molecule belonging to the tetrahydrotriazine-containing novel class of oral glucose-lowering agents (40), suggesting that inhibition of this RET-mediated ROS production may play a role in the mechanisms of action of the two antidiabetic drugs, notably by conferring protection against oxidative stress-related cell death (15, 28, 29). In line with this, a new generation of oxidative stress inhibitors that specifically neutralize ROS produced *via* RET at the I_Q_ site within complex 1 has been shown to lower oxidative damage, inhibit cellular stress signaling and protect against ischemia-reperfusion heart injury (41). Furthermore, it has also been suggested that some of the anti-inflammatory effect of metformin observed in lipopolysaccharide-stimulated bone-marrow derived macrophages could result from the specific inhibition of RET-derived ROS production at the complex 1 (42). Taken together, these findings suggest that targeting RET-linked ROS occurring at the mitochondrial respiratory-chain complex 1 using metformin or metformin-like molecules might be therapeutically relevant in the context of both cardiometabolic and inflammatory diseases.

## Mitochondrial Effects of Metformin and Regulation of Hepatic Gluconeogenesis

Metformin exerts its anti-hyperglycemic action primarily through reduction of hepatic glucose production (3). A major breakthrough occurred in 2001 when Zhou and colleagues reported that metformin increased the AMP-activated protein kinase (AMPK) activity in hepatocytes, a feature associated with inhibition of gluconeogenesis (43). AMPK is a protein kinase that functions as energy gauge which constantly senses the cellular energy status by monitoring AMP, ADP, and ATP levels (44, 45). Once activated in response to decrease in ATP and concomitant rise in intracellular ADP and AMP levels, AMPK inhibits ATP-consuming anabolic processes and promotes ATP-generating catabolic pathways by direct phosphorylation of a broad range of downstream effectors that are involved in the regulation of various metabolic processes, ultimately leading to restoration of cellular energy balance (44). It took a decade of controversy before the general acceptance that AMPK activation by metformin results from increased ADP:ATP and AMP:ATP ratios secondary to inhibition of the mitochondrial respiratory-chain complex 1 (3). Of note, only biguanides with physicochemical characteristics allowing them to enter the mitochondria and to inhibit complex 1 were shown to activate AMPK (32). However, using liver-specific AMPK knockout mice, Foretz and colleagues demonstrated that metformin lowers gluconeogenesis by an AMPK-independent mechanism involving a decrease in cellular energy state, a strong correlation being observed between the increase in cellular [AMP]:[ATP] and the inhibition of gluconeogenesis (46). Altogether, although metformin can activate AMPK, it is therefore neither necessary nor sufficient for inducing acute inhibition of gluconeogenesis (Figure 1). In line with this, two other studies also demonstrated that metformin can inhibit hepatic glucose production through AMPK-independent mechanisms: one by AMP-mediated inhibition of adenylate cyclase and subsequent reduction in glucagon-increased cyclic adenosine monophosphate (cAMP) levels (47); the other one through modulation of cytosolic redox state *via* direct inhibition of the mitochondrial glycerol-3-phosphate dehydrogenase (mGP_DH_) (8) (Figure 1). mGP_DH_ is a flavin-linked respiratory-chain dehydrogenase belonging to the glycerol phosphate shuttle that couples the oxidation of glycerol-3-phosphate to dihydroxyacetone with reduction of FAD to FADH_2_ and the transfer of electrons to coenzyme Q of the ETC, contributing as such to the maintenance of the redox potential across the inner mitochondrial membrane (48). Remarkably, Shulman's group reported that metformin exerts an *in vitro* non-competitive inhibition of the enzyme, with a K_i_ value (~40 μM) within the clinical range of drug concentrations, leading to increased hepatic cytosolic NADH/NAD^+^ ratio and impaired gluconeogenesis from redox-dependent substrates, such as lactate and glycerol, in rats (8). In a recent follow-up study, they showed that both acute and chronic treatment with metformin also inhibit hepatic gluconeogenesis in a redox-dependent manner in diabetic rats, without apparent changes in mitochondrial citrate synthase flux and hepatic nucleotide concentrations (49). By contrast, Sakamoto's group recently provided new supportive elements strengthening the key role of mitochondria-mediated modulation of cellular energy homeostasis in the inhibition of hepatic gluconeogenesis by metformin. Indeed, in an elegant study using knock-in mice, the authors demonstrated that a point mutation in the gluconeogenic enzyme fructose-1,6-bisphosphatase-1 (F1BP) which impairs its allosteric inhibition by AMP reduced the anti-hyperglycemic effect of metformin in diabetic mice (50). Altogether, this strongly suggests that the transient rise in intracellular AMP levels resulting from the weak and reversible inhibition of the respiratory-chain complex 1 by metformin is crucial for inhibiting hepatic gluconeogenesis, either by modulating adenylate cyclase or FBP1 activity (Figure 1).

## Inhibition of Complex 1 by Metformin and Metabolic Reprogramming in Cancer Cells

A growing body of epidemiological and clinical studies reported that metformin reduces cancer risk in patients with type 2 diabetes and improves survival outcome of cancer patients with breast, ovarian, liver and colorectal tumors (51). Although an extensive overview on this topic can be found elsewhere [for recent reviews see (51–54)], it is striking that the mitochondrial effect of metformin could again play a crucial role in the anti-tumorigenic effect of the drug. Indeed, the inhibition of complex 1 was observed in many cancer cells (Table 1) and usually leads to reduced mitochondrial OXPHOS and ATP depletion, ultimately resulting in AMPK-mediated activation of catabolic pathways and inhibition of anabolic processes through its regulation of mechanistic target of rapamycin complex 1 (mTORC1) (54). While some AMPK- and mTORC1-independent mechanisms can also co-exist (55), this metabolic reprogramming lowers growth and proliferation of cancer cells, at least partly due to inhibition of protein and lipid synthesis. It also promotes cell cycle arrest and apoptosis in cells that cannot cope with the energetic stress (54). Wheaton and colleagues clearly showed that the reduction of tumor growth by metformin was prevented in cancer cells expressing NDI1, a metformin-resistant yeast analog of complex 1, highlighting the central role played by inhibition of this mitochondrial target in the antineoplastic effect of the drug (21). This is also consistent with another study showing that phenformin exerts its anti-tumorigenic effects by inhibiting complex 1 (56). Nevertheless, most of the effects of metformin were generally observed at supratherapeutic concentrations and the drug bioavailability in cancer cells is still questionable. Interestingly, more lipophilic derivatives of metformin targeting the mitochondria are currently under investigation with the aim of developing analogs with higher bioavailability and antitumor activity than metformin. Remarkably, some of these newly synthetized molecules were recently reported to be nearly 1,000-fold more potent than metformin in inhibiting mitochondrial complex 1 activity and to exert both anti-proliferative and radiosensitizing effects in pancreatic cancer cells (23). Altogether, developing such kind of mitochondria-targeted metformin-like drugs could pave the way for promising new therapeutic strategies that might also be relevant for various other pathologies than cancer (57).

## Concluding Remarks

Although the interest around metformin has been significantly revived during the last years, principally due to the potential repositioning of this antidiabetic drug for the treatment of cancer, it still remains crucial to better decipher the mechanism by which it inhibits the mitochondrial respiratory-chain complex 1, notably the exact nature of their interaction. Elucidating this aspect may advance our understanding of how metformin regulates cellular energetics and be decisive for optimizing future drug development and therapeutic interventions, notably for cancer patients.

## Author Contributions

GV performed literature search and drafted the manuscript. DD performed literature search and drafted the manuscript. BG performed literature search, designed the figure, wrote, and edited the manuscript.

### Conflict of Interest Statement

The authors declare that the research was conducted in the absence of any commercial or financial relationships that could be construed as a potential conflict of interest.
